# DRLBTS: deep reinforcement learning-aware blockchain-based healthcare system

**DOI:** 10.1038/s41598-023-29170-2

**Published:** 2023-03-13

**Authors:** Abdullah Lakhan, Mazin Abed Mohammed, Jan Nedoma, Radek Martinek, Prayag Tiwari, Neeraj Kumar

**Affiliations:** 1 Department of Computer Science, Dawood University of Engineering and Technology, Sindh, Karachi, 74800 Pakistan; 2 College of Computer Science and Information Technology, University of Anbar, Anbar, 31001 Iraq; 3 Department of Telecommunications, VSB-Technical University of Ostrava, 70800 Ostrava, Czech Republic; 4 Department of Cybernetics and Biomedical Engineering, VSB-Technical University of Ostrava, 70800 Ostrava, Czech Republic; 5 School of Information Technology, Halmstad University, Halmstad, Sweden; 6 Department of Computer Science and Engineering, Thapar Institute of Engineering and Technology (Deemed University), Patiala, Punjab India; 7 School of Computer Science, University of Petroleum and Energy Studies, Dehradun, Uttarakhand India; 8 Department of Computer Science and Information Engineering, Asia University, Taichung, Taiwan

**Keywords:** Computer science, Bioinformatics

## Abstract

Industrial Internet of Things (IIoT) is the new paradigm to perform different healthcare  applications with different services in daily life. Healthcare applications based on IIoT paradigm are widely used to track patients health status using remote healthcare technologies. Complex biomedical sensors exploit wireless technologies, and remote services in terms of industrial workflow applications to perform different healthcare tasks, such as like heartbeat, blood pressure and others. However, existing industrial healthcare technoloiges still has to deal with many problems, such as security, task scheduling, and the cost of processing tasks in IIoT based healthcare paradigms. This paper proposes a new solution to the above-mentioned issues and presents the deep reinforcement learning-aware blockchain-based task scheduling (DRLBTS) algorithm framework with different goals. DRLBTS provides security and makespan efficient scheduling for the healthcare applications. Then, it shares secure and valid data between connected network nodes after the initial assignment and data validation. Statistical results show that DRLBTS is adaptive and meets the security, privacy, and makespan requirements of healthcare applications in the distributed network.

## Introduction

The usage IIoT paradigm based on machine learning schemes have been rising day by day^1^, the goal is to offer different automated machine learning enabled healthcare services to users. The digital healthcare applications such as COVID-19 detection systems, heartbeat monitoring systems, cancer detection systems, and many other applications executed on distributed IIoT networks^2^. The IIoT network is made up of different technologies, such as IoT devices (like healthcare sensors), blockchain technologies, different wireless technologies (e.g., Bluetooth and 5G/6G technologies), and cloud computing (e.g., fog nodes and edge nodes). These technologies are exploited to run the IIoT healthcare applications based on above mentioned technologies^3^. Task scheduling is the most important mechanism of IIoT paradigm for healthcare applications to meet their quality of service needs during execution in different technologies. There are different types of healthcare applications, such as workflow, coarse-grained, and object-based Fine-grained tasks. On the other hand, all medical services are carried out according to a sequential workflow pattern. Because of this, it is hard to schedule healthcare workflow applications based on the quality of service they need in the IIoT paradigm. Generally, healthcare workflow applications are scheduled on cloud computing-based resources distributed across different networks. Static scheduling causes significant problems in healthcare applications. For example, the assignment of healthcare workflows to cloud resources by static schedulers cannot be changed if the performance of a healthcare application degrades while it is running. Dynamic scheduling is a good way to make changes during the execution of a healthcare workflow application on the cloud. Regardless, dynamic schedulers continue to fail due to the limited capacity of edge computing resources for applications. 

To solve the workflow healthcare application scheduling problems in distributed cloud computing, many adaptive task scheduling schemes have been suggested in the literature. In the literature, many supervised and unsupervised learning-based scheduling approaches are suggested to predict and run workflows quality of service requirements. The reinforcement learning based scheduler optimizes the healthcare application performance with q-learning policies and value functions. The IIoT paradigm based healthcare problem is not like the game theory problem or the factory automation problem, but it is a dynamic problem with the data. However, these healthcare applications are distributed and offload their data to various independent nodes, security issues are the primary challenges in decentralized uniform nodes.

Recently, many IIoT paradigm based applications have been implemented based on decentralized blockchain technology for independent nodes, where data tampering and security issues are somehow reduced in the network^4^. Blockchain technology has different types: public, private, consortium, and community. Ethereum, Corda, Hyper-ledger, IBM, and many more are all public frameworks for blockchain technology that is already in use. Many machine learning models are suggested with blockchain technology to control the load balancing between resources and energy consumption in the IoT network^5,6^. Deep learning is a type of machine learning in which we can use different artificial neural networks to improve a lot of IoT system metrics. However, all existing machine learning-enabled industrial IoT systems support only financial applications. The healthcare applications are widely ignored in the literature^6–8^.

These workloads, such as fine-grained objects, and cluster workload are deployed in the healthcare system. These workloads  are set up based on an IoT fog cloud, that reduces the  processing time. The current IIoT paradigm has different types, like the Internet of Medical Things (IoMT) and Industrial Internet of Healthcare Things (IoHT), these paradigm only assumes the workloads mentioned earlier on the edge cloud network with different constraints (like delay, energy, tardiness, and resources). The existing IoMT and IoHT systems use blockchain technology and hasn’t paid much attention to workflow applications, which run in different computing nodes due to limited resources.

The single agent-based reinforcement learning approaches suggested in these studies^9,10^. The goal was to use trial and error techniques with dynamic gain to improve the performance of distributed applications. A few studies suggested multi-agent-based policies with the cooperative nodes in the network^11,12^. The goal is to transfer workflow based on their policies for predicting time series and get the best reward for a given discount factor. Because there are more workloads and nodes, the current multi-agent with deep reinforcement learning and the q-learning policy has more variation and delay time. Distributed applications can run into trouble when multi-agent policies with security constraints, deadline constraints, cost constraints, and delay constraints are in place.

 This paper represents an algorithm framework for healthcare workflow applications called DRLBTS (deep reinforcement learning and blockchain-enabled task scheduling). DRLBTS consists of different schemes, such as Q-learning, blockchain, and task scheduling. The goal is to minimize the makespan of the workflow applications. The makespan is the combination of the communication time and computation on different nodes (e.g., mobile, fog, and cloud) during processing. The paper makes the following to the considered workflow scheduling problem in heterogeneous computing nodes.

- (1) we design the system based on three types of processing nodes: mobile, fog, and cloud nodes. The goal is to run different workflow tasks, such as lightweight, delay and delay tolerant tasks on different nodes.
- (2) The system provide the public blockchain scheme to maintain the data processing workflow of healthcare applications across multiple computing nodes.
- (3) The application partitioning and resource profiling schemes are designed to maintain resource balancing during processing workflows on computing nodes.
- (4) Based on their current state and their reward, the study creates an agent policy that helps move work from mobile devices to collaborative fog nodes and the cloud. The work implements the delay optimal deep reinforcement learning scheme, whereas adaptive scheduling is devised based on q-learning schemes.
- (5) The designed model is lightweight proof of work (PoW), focusing on data validity, security, and efficient processing among different nodes.
- (6) The study also provide a mathematical model, in which workflow task assignment, processing time, and communication time are determined by different equations. The objective function and constraints are analyzed in the mathematical model.

The manuscript is divided into sub-parts to define the different steps of the work. In related work, there is a lot of talk about the current deep reinforcement learning methods and blockchain schemes, as well as their solutions and limits. The proposed DRLBTS illustrates how the system works to solve the problem. The DRLBTS algorithm part demonstrates how to solve the problem with different schemes in a given number of steps. The graphs and results of the proposed methods are compared to the baseline methods already in place. This is done to talk about evaluation and implementation. In conclusion, the study’s accomplishments, practices, problems, results, and future work were discussed.

## Related work

 Many studies exploited reinforcement learning, deep reinforcement learning, and blockchain schemes for IIoT paradigm enabled healthcare applications. The study^1^ presented the deep reinforcement learning-based scheme for IIoT healthcare applications in the fog and cloud network. In the study, a single agent was made based on neural network, reinforcement learning, and a prediction of the state and time series in the network. The study^2^  presented the deep reinforcement learning-enabled system for healthcare workloads to optimize the assignment problem. The objective was to optimize resource constraints and allocation constraints in the study. These studies^3–5^ investigated the resource allocation problems for healthcare applications to handle the security issues in the mobile cloud network. These studies devised blockchain technology to handle the data validation between nodes. In this work, the time series prediction and descent gradient-enabled weights were used to meet the needs of the applications. The dynamic allocation of workload based on reinforcement learning with the supervised learning label was investigated by these studies^6–9^. These studies reducing noise by turning unstructured data into structured data. The dynamic task scheduling is based on machine learning and blockchain approaches investigated in these studies^10–15^. These studies devised task-scheduling methods based on different reinforcement learning with different features. The unsupervised learning clustering technique is used to analyze and group together similar types of resources so that tasks that require a lot of data or processing power can be run on them. The aggregation time of resource clustering was determined in these studies. However, scheduling and waiting time were and blockchain security processing time widely ignored in these studies. In these studies, the single-agent scheduling scheme was looked at. In this scheme, data is stored in a different state, and the current state learns from the previous state. The stochastic descent gradient is based on the reinforcement learning task scheduling scheme suggested in these studies ^16–20^, where the heterogeneous cloud nodes (e.g., fog and cloud nodes) and internet of things medical devices are considered in the study. The objective was to optimize the deadline and resource constraints of the devices. The blockchain-enabled task scheduling is based on supervised learning schemes suggested in these studies^21–25^. In the decentralized cloud of the Internet of Things (IoT), proof of work and proof of stake methods are suggested. The data validation at each node matches the hashing and makes immutable network transactions. These algorithms are AES, RSA, MD5, SHA-256^26–31^ implemented in blockchain technology to validate the data between IoT nodes and cloud nodes in the distributed network.

Recently, many studies^31–35^ suggested blockchain enabled deep reinforcement learning schemes with fixed states for healthcare  applications. The goal is to run the healthcare workloads on the fixed processing nodes and improve the reward scores for the applications. The blockchain-enabled homomorphic security and privacy schemes are suggested in these studies^36–40^. These studies implemented blockchain systems that have smart contracts and distributed mining processes to execute fine-grained and coarse-grained healthcare workloads  in the distributed fog cloud networks. All studies investigated task scheduling problems with fine-grained and coarse-grained healthcare workloads. In these studies, the delay, time, deadline, and security validation constraints were considered when the problem was set up. However, these studies should have considered the workflow of healthcare applications on mobile devices. The applications for mobile workflow are complicated and must run on different nodes, like the fog node and the cloud. The main reason is that mobile workflow applications are made up of small tasks that take a lot of processing power and must be run on different nodes. So, deadlines, costs, security, and the possibility of failure are all very important regarding workflow applications that run on different nodes.

 Is a new paradigm of the healthcare application that uses data from different clinics to provide complex remote digital healthcare services to the patients. The IoHT consists of complex biomedical sensors, wireless technology, and cloud computing services that can measure heartbeats, blood pressure, and other things. Industrial healthcare still has to deal with many problems, such as security, task scheduling, and the cost of processing in the cloud. This paper presents a new solution to the issues mentioned above. Existing reinforcement learning and deep learning methods took all the layers and parameters into account, but they were too time-consuming. Also, security is fundamental when different clinics that work independently but in different critical work together on data to make disease prediction and patient applications more efficient. Existing security systems only look at centralized and similar nodes, but when data is moved between clouds and fog, there are security issues. The study devises a deep reinforcement learning-enabled blockchain-based task scheduling (DRLBTS) with many goals. DRLBTS provides efficient scheduling in terms of makespan and meeting the requirements of healthcare workflow applications in the mobile fog cloud networks. Shares secure and valid data between connected network nodes. The proposed q-learning-based scheduling executes all workflow applications until and unless they meet their requirements in the mobile fog cloud networks.

## Proposed DRLBTS system

**Figure 1:**
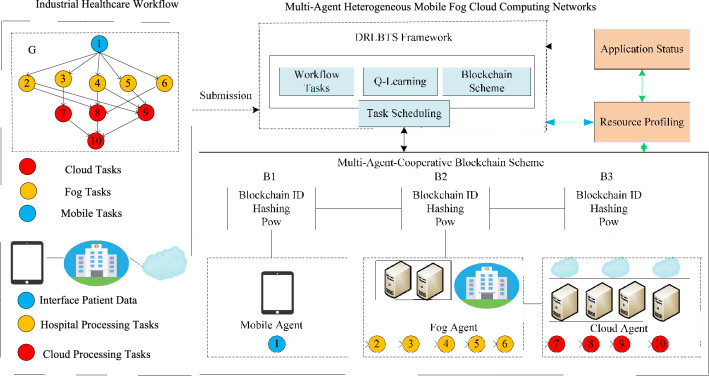
DRLBTS: deep reinforcement learning-aware blockchain-based healthcare system.

 The study presents a deep reinforcement learning-aware blockchain-based healthcare system, as shown in Fig. 1. The study considers the industrial healthcare workflow, which is a single workflow, e.g., v1=1,...v=10. The study partitioned the workflow application at the design time into mobile, fog, and cloud tasks. The main reason is that the mobile device doesn't have enough resources to handle all tasks, fog nodes can only handle delay-sensitive tasks, and cloud computing can handle delay-tolerant tasks. The study differentiates tasks based on their annotations. For instance, blue nodes show mobile tasks, yellow nodes show fog tasks, and red nodes show cloud tasks. The patients' interfaces are their mobile devices, which they can use to request or upload data in the workflow pattern. The hospital's processing tasks will be executed on the system's fog node. The cloud processing tasks will store their huge data after execution on the cloud node. The multi-agent heterogeneous mobile fog cloud networks demonstrate that workflow application tasks in a workflow pattern should be performed on different nodes. The mobile agent, for example, completes task 1, the fog agent completes tasks 2, 3, 4, 5, and 6, and the cloud agent completes tasks 7, 8, 9, and 10. The DRLBTS framework comprises different plans, like task scheduling, q-learning, blockchain schemes, and workflow task sequencing. All the computing nodes are cooperative and share the data. All the nodes are connected based on the blockchain network, where each piece of data is converted into a hash and shared in a valid format. B stands for the blockchain blocks, each of which has a unique identification number (ID). Each task transaction must be verified using the PoW scheme. Application status and resource profiling are schemes that notify the system about the entire execution of the workflow tasks on the mobile fog cloud network.

### Electronic health record (EHR)

In our scenario, EHR consisted of mobile patient  application where hospital services integrated at the smart-city and global levels based on fog and cloud networks. The mobile patients can access city level services by fog nodes. Furthermore, few services are globally available based on cloud computing in the entire region for healthcare application. The study considered the workflow of healthcare applications and exploited them on different nodes such as mobile, fog, and cloud. To ensure security, the study implemented blockchain schemes and performed valid data transactions among nodes.

### Problem mathematical model of mobile-fog-cloud agents

In this paper, the mobile workflow is represented by the workflow, e.g., (*G,V* ), where the V set of workflow tasks consists of mobile, fog, and cloud tasks. There is an *A* number of workflows in this work. *E* is the communication weightage between local, fog, and cloud tasks. The workflow is broken down into three sub-task sets: *mobile*[*v* = 1 V ], and *cloud*[*v* = 1 V ]. Each workflow has data, like *data*_*V*_ , broken up into subtasks and handled by the different agents. The investigation is centered on three computing nodes: mobile agents *m*, fog nodes *f* , and cloud nodes *c*. Each node, such as mobile *m, fog node f, and cloud node c*, has a particular speed and resources in the network, for instance, *epsilon*_*m*_*, epsilon*_*f*_* , andepsilon*_*c*_, for example, represent the resources of mobile agents, fog agents, and cloud agents in the network, with their respective speeds, e.g., ${\zeta_{m}}$*, *${\zeta_{f}}$, ${\zeta_{c}}$. Three kinds of agents can run workflow applications based on how their tasks were marked up when the applications were being made.

1 $$T_{v}^{e} = \begin{array}{*{20}c} {\frac{{mobile_{v = 1 \in \;V} \in G(data_{v} )}}{{\zeta_{m} }},\quad y_{v} = 1, } \\ {\frac{{fog_{v = 1 \in \;V} \in G(data_{v} )}}{{\zeta_{f} }},\quad y_{v} = 2, } \\ {\frac{{cloud_{v = 1 \in \;V} \in G(data_{v} )}}{{\zeta_{c} }},\quad y_{v} = 3.} \\ \end{array}$$

Equation (1) determines the execution time of a set of tasks on the different nodes. Whereas, *y*_*v*_ = 1*,* 2*,* 3 is the assignment vector which shows that, either task are assigned or not to the particular mobile, fog, and cloud nodes. The study determines the communication time of workflow tasks in the following way.

2 $$E_{v1,v2} = \begin{array}{*{20}c} {\frac{{mobile_{v = 1 \in \;V} \in G(data_{v} )}}{{bw_{m} }},\quad x_{v1,v2} = 1, } \\ {\frac{{fog_{v = 1 \in \;V} \in G(data_{v} )}}{{bw_{f} }},\quad x_{v1,v2} = 2, } \\ {\frac{{cloud_{v = 1 \in \;V} \in G(data_{v} )}}{{bw_{c} }},\quad x_{v1,v2} = 3.} \\ \end{array}$$

Equation (2) determines the communication between the mobile node and the fog node, and the fog node’s proximity to the cloud node determines the communication. We assume all nodes have fixed data communication bandwidth in the network.

3 $$S_{s1,a1} = \begin{array}{*{20}c} {B \leftarrow data_{v = 1 \in \;V} ,\quad s_{s1,a1} = 1, } \\ {B \leftarrow data_{v = 1 \in \;V} ,\quad s_{s2,a2}^{\prime } = 2, } \\ {B \leftarrow data_{v = 1 \in \;V} ,\quad s_{s3,a3}^{\prime \prime } = 3.} \\ \end{array}$$

The State Equation (3) determines that each state has a complete execution process and a blockchain scheme to transfer workflow between tasks. The total execution time of all workflow applications is determined below.

4 $$X = \sum\limits_{G = 1}^{A} {\sum\limits_{v = 1}^{V} {T_{v}^{e} + E_{v1,v2} + S_{a1,s1} } } ,\quad {\text{m,}}\;{\text{f,}}\;{\text{c}}\;{\text{nodes}}{.}$$

Equation (4) determines the makespan time of all workflow applications at the different computing nodes for the considered problem. The study is to be optimized for the objective function of the study in the following Eq. (5).

5 $$\begin{array}{*{20}c} {\min \quad X} \\ {{\text{Subject}}\;{\text{to}}} \\ \end{array}$$

6 $$\sum\limits_{G = 1}^{A} {mobile[v = 1 \in V] \leftarrow data_{v} \le \varepsilon_{f} } , \quad \forall G = 1, \ldots ,A.$$

Equation (6) illustrates that all the local tasks must be executed under the resource limit of mobile nodes in the network.

7 $$\sum\limits_{G = 1}^{A} {fog[v = 1 \in V] \leftarrow data_{v} \le \varepsilon_{m} } , \quad \forall G = 1, \ldots ,A.$$

Equation (7) illustrates that all the fog tasks must be executed under the resource limit of fog nodes in the network.

8 $$\sum\limits_{G = 1}^{A} {cloud[v = 1 \in V] \leftarrow data_{v} \le \varepsilon_{c} } , \quad \forall G = 1, \ldots ,A.$$

Equation (8) illustrates that all the cloud tasks must be executed under the resource limit of cloud nodes in the network. Each workflow has a deadline, e.g., *D*_*G*_, and has the following constraint.

9 $$\sum\limits_{G = 1}^{A} {T^{e} [v = 1 \in V] \leftarrow data_{v} \le D_{G} } , \quad \forall G = 1, \ldots ,A.$$

The deadline Eq. (9) determines that all workflow applications must be executed under their deadline bias in the network.

### DRLBTS algorithm methods

The study presents the DRLBTS algorithm framework to solve the task scheduling problem on the different computing nodes for the workflows. DRLBTS consists of different schemes to solve the problem in different steps. Initially, DRLBTS introduces the deep q-learning approach where tasks are divided into different categories as shown in Algorithm 1. Furthermore, Algorithm 1 consists of different schemes to show the execution of applications with their workflows. The divided tasks are distributed among mobile, fog, and cloud-based systems based on their annotations. The annotations are types of tasks executed on mobile, fog, and cloud tasks, decided by the application’s partitioning scheme in the design time of the system. All tasks are scheduled on computing nodes based on their types. However, with adaptive q-learning enabled scheduling handles all failure tasks and reschedule them on the available resources computing nodes.
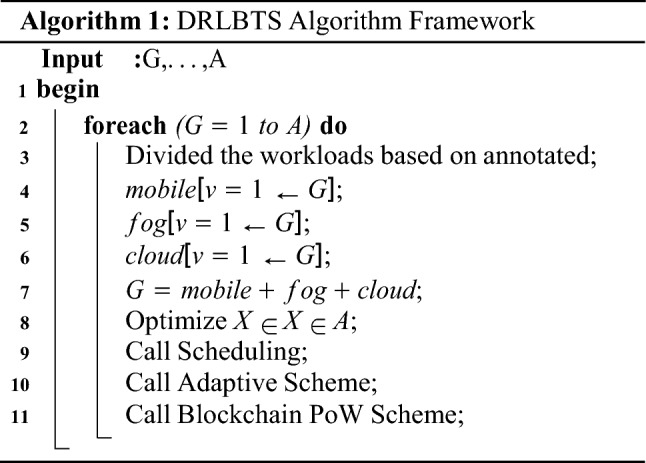


### Deep reinforcement learning multi-agents

The considered task scheduling problem has a finite loop with different states, the optimal policy and value function are examined the execution performance of workflow tasks. In our study, the deep reinforcement learning method is a combination of two different approaches, such as deep learning and reinforcement learning. The main reason is that we obtained the results of running applications through trial and error parameters because the availability of computing resources change all the time. We use supervised learning to label all of the inputs in advance, such as *mobile*[*v* = $\text{v} \in V$], *f og*[*v* = $\text{v} \in V$], and *f og*[*v* = $\text{v} \in V$]. The study considers multi-agent systems where each agent can communicate with another agent to perform tasks and share valid task data for further execution. The study initially divided the deadlines of all workflows according to their makespan in the following way.

10 $$\sum\limits_{v = 1}^{V} {\sum\limits_{G = 1}^{A} {d_{v} = D_{G} - T_{v}^{e} + E_{v1.v2} - mobile[v = 1 \in V] + fog[v = 1 \in V] + cloud[v = 1 \in V].} }$$

Equation (10) determines the deadline of each task based on its execution time. The probability of the study is determined in the following

11 $$\sum\limits_{v = 1}^{V} {\sum\limits_{G = 1}^{A} {P(s,a,t||s^{\prime } ||s^{\prime \prime } ).} }$$

whereas *t* is the timestamp of the state transition in work. The equation (11) determines the probability of each agent. The policy of the objective function *X* determines the following.

12 $$\pi = X \leftarrow (s|a).$$

Equation (12) determines the optimal policy of the workflows in different network states.

### Policy enabled workflows assignment in different agents

The Algorithm 2 demonstrates that all agents, such as mobile, fog, and cloud, must execute their tasks in their respective states with the optimal policy and objective in work. In a mobile cloud network, each state must meet the deadline, security, and makespan of the workflows between the different pieces of data. All the agents are cooperative and share their data between tasks. The main reason is that, the system executes the tasks of workflow applications on different nodes. Therefore, the data must be shared between tasks on the different nodes.
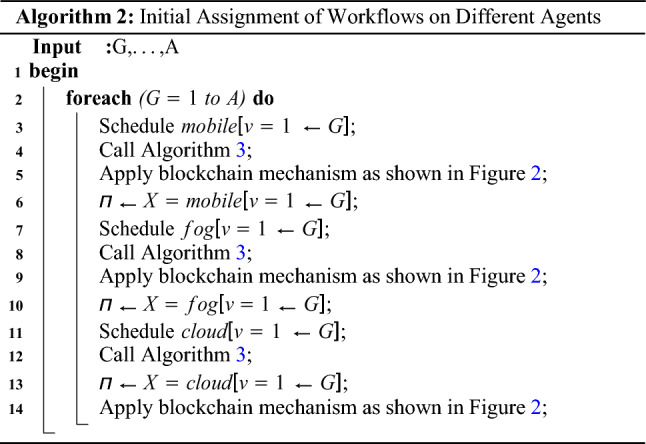


### Adaptive task scheduling and blockchain mechanism

**Figure 2:**
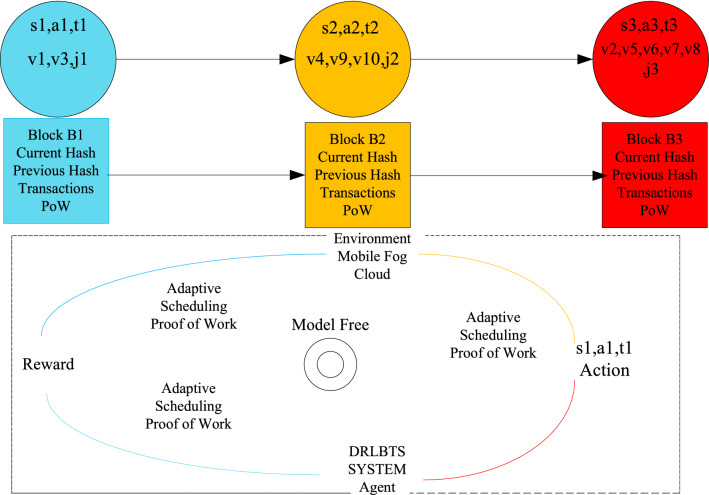
Deep-reinforcement learning-enabled blockchain mechanism.

Blockchain-enabled enabled scheme and adaptive scheduling scheme maintains the performance of workflow tasks among different agent states illustrated in Fig. 2. At the time of design, all tasks are split up into those done on the local machine and those done on the fog node and cloud. This is shown in Fig. 2. At the same time, the execution was divided into different states and ran in other time transitions with the policy and reward constraint. Algorithm 3 is the adaptive scheduler based on blockchain technology in work. The scheduling divides into different states when optimizing the objective function of the workflow applications. In the initial state, all the mobile tasks are scheduled on the mobile device according to mobile resources and speed with the state, action, and transition. Algorithm 3 has the following execution process with different steps.
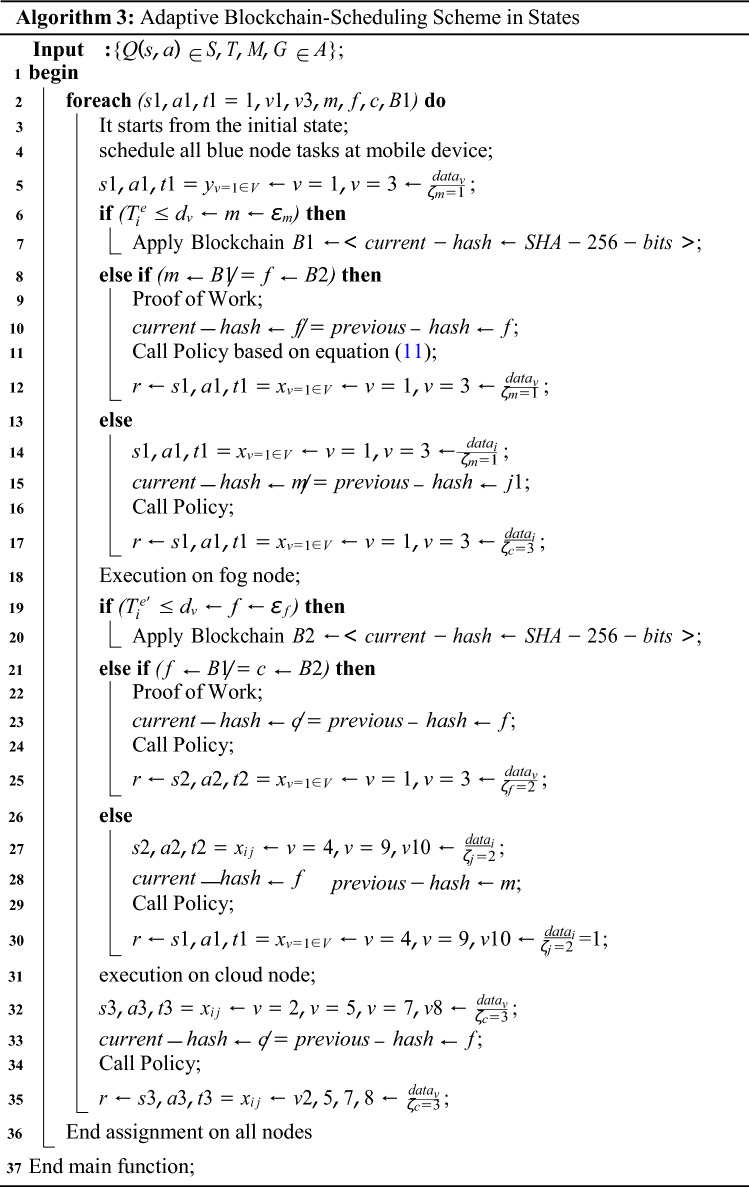

- Mobile Execution: In the schedule from steps 1 to 10, all the designed annotated local schedules at the mobile device and applied the blockchain validation in the mobile device. The initial state from *s*1*, a*1*, t*1 is the first action and transition in the state. In this state, for the application, only *v*1*, v*3 tasks are scheduled on the mobile node *j*1. This process will encrypt based on Secure hashing algorithm (SHA-256) bits, and cryptographic data offload from one *j*1 to *j*2 until and unless the current and previous hash is matched. The model-free optimal policy call optimizes and adds the reward in the q-learning sequence with the positive execution. The process of mobile task execution is represented in Fig. 2.
- Fog Execution: In the schedule from steps 11 to 23, all the designed annotated yellow tasks schedule at the fog node, and apply the blockchain validation in the fog node. The initial state from *s*2*, a*2*, t*2 is the first action and transition in the state. In this state, for the application, only *v*4*, v*9*, v*10 tasks are scheduled on the fog node *j*2. This process will be encrypted based on Secure hashing algorithm (SHA-256) bits, and cryptographic data offload from one *j*2 to *j*3 until and unless the current and previous hash matches. The model-free optimal policy call optimizes and adds the reward in the q-learning sequence with the positive execution. The process of mobile task execution is represented in Fig. 2.
- Cloud Execution: In the schedule from steps 24 to 35, all the designed red annotated cloud schedule tasks at the mobile cloud node and apply the blockchain validation in the cloud computing. The initial state from *s*3*, a*3*, t*3 is the first action and transition in the state. In this state, for the application, only *v*2*, v*5*, v*6*, v*7*, v*8 tasks are scheduled on the mobile node *j*3. This process will be encrypted based on Secure hashing algorithm (SHA-256) bits, and cryptographic data offload from one *j*3 to *j*2 until and unless the current and previous hash matches. The model-free optimal policy call optimizes and adds the reward in the q-learning sequence with the positive execution. The process of cloud task execution is represented in Fig. 2.

### Evaluation and implementation part

 In this session, we discuss the implementation of DRLBTS and baseline algorithms and their performance in result discuss part.  The experimental parameters exploited in the implementation are defined in Table 1. The study compares the obtained data results based on the statistical mean values by the relative percentage deviation as shown in Eq. (13). Whereas, X represents the objective function of the study, and X* is the obtained optimal objectives in the adaptive scheduling. RPD% differentiates between optimal and objective function and initial obtained objective function of the workflow applications by the adaptive scheduling. 

13 $$RPD\% = \frac{{X - X^{*} }}{{X^{*} }}100\% .$$

**Table 1:** Experimental parameter setting.

Notation	Description	Configuration
*j*1	Vivo-15 Android Mobile	Config File
*ζ*_*j*_	8 GB Ram	Config File
*ε j*1	64 GB Rom	Config File
*j*2	Core i7 Laptop Device Fog node	Config File
*ζ*_*j*_	16 GB Ram	Config File
*ε j*1	500 GB Rom	Config File
*ζ*_*j*_	16 GB Ram	Config File
*ε j*1	500 GB Rom	Config File
*j*3	Dual High processing Amazon Cloud	Config File
*G*1	https://www.kaggle.com/shayanfazeli/heartbeat	Infrastructure file
*V* = 1000 ← *G*1 *G*2	Number of workflow tasks https://www.kaggle.com/Heartbeat-Workflow-general	Infrastructure file Infrastructure file
*V* = 800 ← *G*2	Number of workflow tasks	Infrastructure file
*G*3	https://www.kaggle.com/general/229244-1255772	Infrastructure file
*V* = 600 ← *G*3	Number of workflow tasks	Infrastructure file
*G*4	https://www.kaggle.com/heartbeat-workflow-general/229244-1255772	Infrastructure file
*V* = 700 ← *G*4	Number of workflow tasks	Infrastructure file
*S*	Number of states: 20	Infrastructure file
*a*	Number of actions: 20	Infrastructure file
*t*	Number of transition: 20	Infrastructure file
*N*	Number of blockchain blocks: 5	Infrastructure file
*B*	Particular blockchain block	Infrastructure file
*SHA* − 256*bits*	Encryption Scheme	XML File
*upload* − *bandwidth*	600 MB	XML File
*download* − *bandwidth*	600 MB	XML File
Case1:	All execution on local device	Simulation File
Case2:	All execution on Fog Node	Simulation File
Case3:	All execution Cloud node	Simulation File
Case4:	All execution on mobile fog cloud node	Simulation File

Equation (13) shows the statistical analysis of the proposed methods based on the given data with both single variance and multi-variance during simulations.

### Use-cases and baseline approaches of workflow healthcare applications

The study designed the simulation based on different layers, such as industrial healthcare workflow, multi-agent heterogeneous nodes, and mobile fog and cloud agents. The baseline of the code is designed on edgexfoundry where layers can easily implement due to the open-source application programming interface. The industrial workflow applications are divided mobile devices into different parts such as mobile, fog, and cloud tasks.

For the experimental comparison, the study used existing algorithms and workflow workloads, which are described below.

- DQN (Deep Q-Learning) implemented as Baseline 1 is widely approached in the deep reinforcement learning approach to solve the problem of heterogeneous computing. This method is widely exploited in these^1–4^ studies when considering similar workflow problems in different computing nodes.
- DDPG machine learning approach is widely implemented as the Baseline 2 scheme for similar workloads and problems as considered in these studies^5–8^ during problem formulation in networks.
- DDPG with blockchain-enabled approaches implemented as the Baseline 3, which are deployed to improve the dynamic scheduling performances for healthcare applications by these studies^9,10^.
- Asynchronous Advantage Actor-Critic Algorithm and decentralized ethereum scheduling implemented as the Baseline 4 in the simulation of these studies^9,10^. The state search strategy is designed to achieve dynamic uncertainty of resources.

### Parameter variation

Table 2 shows that the variation in states, actions, transition, and blockchain blocks increases resource consumption. In this study, we randomly put the *S* = 20*, a* = 20*, t* = 20*, N* = 5*, B* = 1 5 based on available mobile fog and cloud nodes and two failure transactions during the experiment for each workflow. However, increasing the states, actions, transactions, and blocks will optimize scheduling chances, but resource consumption increases and leads to higher resource leakage.

**Table 2:** Metrics comparison.

G	a	t	N	B	RPD%	Resources
1000	20	20	5	5	82	RAM 2048, CPU 51%
800	20	20	5	5	67	RAM 2048, CPU 50%
600	20	20	5	5	71	RAM 2048, CPU 49%
700	20	20	5	5	77	RAM 2048, CPU 50.5%
1000	25	25	5	5	87	RAM 2048, CPU 56%
800	20	20	5	5	71	RAM 2048, CPU 54%
600	20	20	5	5	78	RAM 2048, CPU 53%
700	20	20	5	5	85	RAM 2048, CPU 55.56%

## Result discussion with different approaches

**Figure 3:**
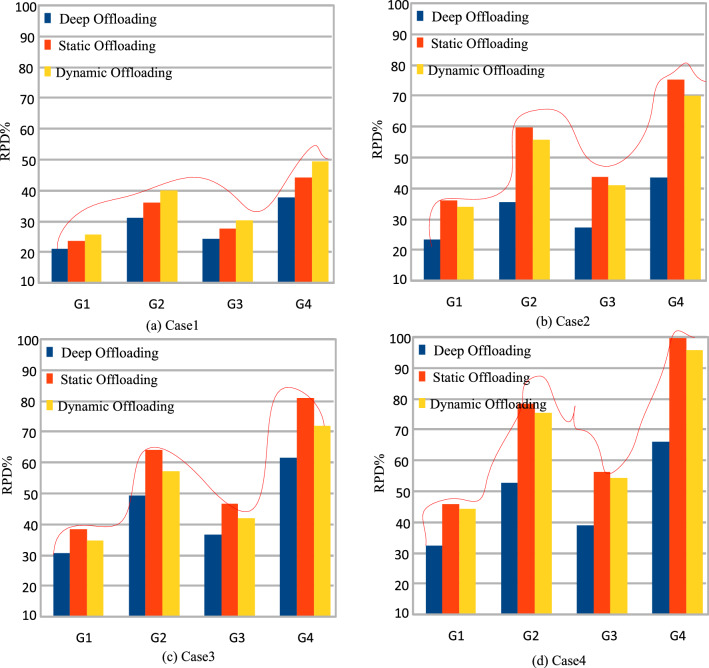
Blockchain enabled validation performance in multi-agent cooperative nodes.

**Figure 4:**
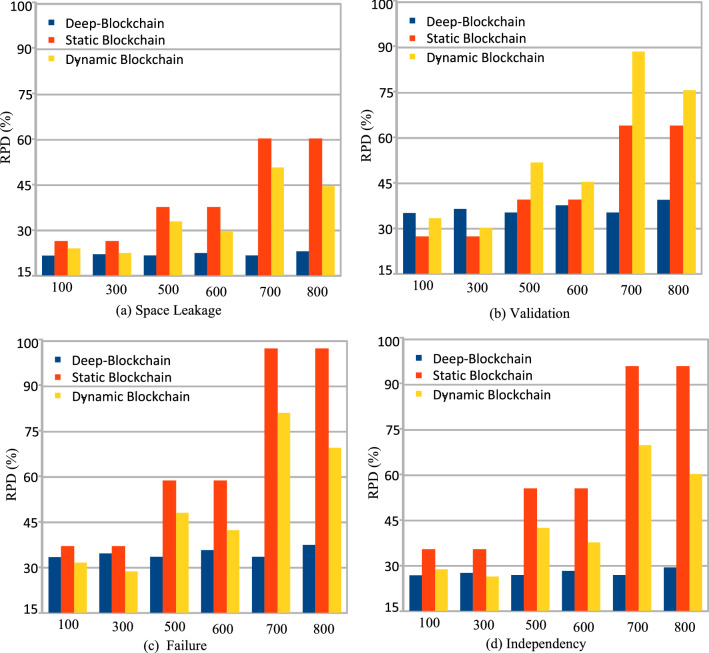
Blockchain-enable data transitions in mobile fog cloud networks.

**Figure 5:**
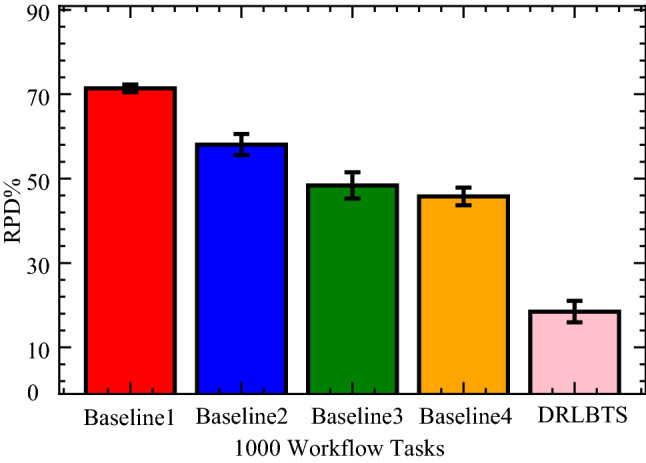
Makespan performance of workflows in mobile fog cloud networks.

**Figure 6:**
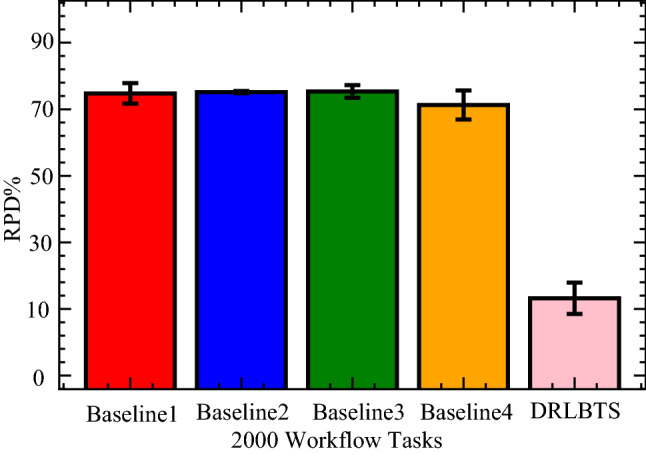
Adaption performance of scheduling schemes for workflows.

**Figure 7:**
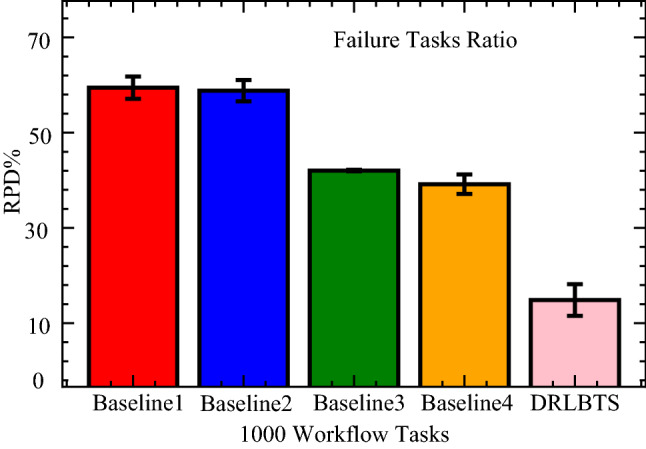
Performance of workflows based on given policy.

**Figure 8:**
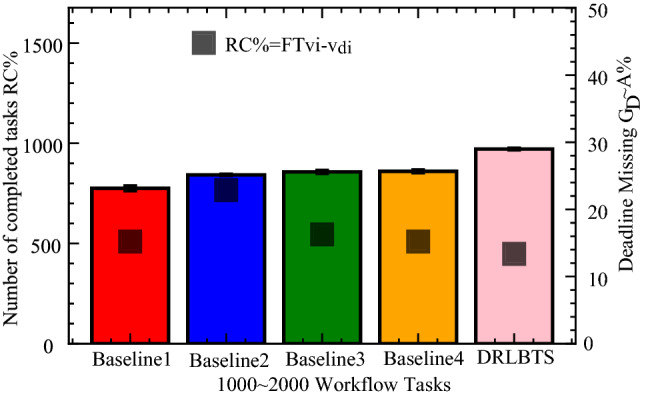
Baseline deep and reinforcement learning schemes for workflows.

In this part, the study analyzes the performance of the different workflow applications on the various computing nodes in four separate cases. In case 1, the study executes all workflow applications on mobile devices, as shown in Fig. 3 (a). Figures (b), (c), and (d) (cases 2, 3, and 4) depict the scheduling and offloading performance of workflow applications in other nodes. But it can be seen that the deep offloading method suggested by the study is better than the methods that are already used to run workflow applications on different nodes. The implemented strategies, such as Deep Offloading, Static Offloading and Dynamic Offloading migrates workload from resource constraints local devices to the available nodes for execution based on given requirements. Figure 3 shows that four applications, such as *G*1*, G*2*, G*3*, G*4 offload their workloads from local devices to proximity computing for execution. At the same time, the y-axis represents the RPD% point values related to the objective function. Figure 3 (a) shows that the simple parameter-enabled static offloading has higher delays when the parameters are changed at the runtime. The considered parameters are resources, traffic, and waiting time during execution, as shown in Fig. 3 (a,b,c,d). Figure 3 (a) shows that the simple parameter-enabled dynamic offloading has higher delays when the parameters are changed at the runtime. The considered parameters are resources, traffic, and waiting time during execution, as shown in Fig. 3 (a,bc,c,d). Dynamic offloading schemes adopt some changes; however, these offloading methods have higher delays due to failure of workflow tasks and unavailability of resources in the particular nodes. Therefore, deep offloading method considered all aspects during processing of workflow on the computing nodes, such as parameters changing, resource and tasks failure, and deadline, therefore it has lower delays as shown in Fig. 3 (a,b,c,d). Figure 4 shows that deep learning-enabled blockchain for the workflow healthcare application is the more optimal in terms of resource leakage, data validation, transaction failure, and inter-dependency as shown in Fig. 4 (a), (b), (c) and (d). The existing static blockchain ethereum and dynamic Corda blockchain did not focus on resource leakage, tasks failure, and workflow dependency in their models. Because these blockchain frameworks only considered the coarse-grained and fine-grained workloads in their models that are executed on the distinct nodes. Figure 4 (a) shows the performance of blockchain technologies during their data validations of healthcare workflows among different computing nodes. However, we determined that there is a considerable delay due to resource-constraint issues among different computing nodes while performing validation on data transactions of healthcare workflows. Furthermore, resource leakage in different computing nodes is the biggest issue while implementing the the existing blockchain technologies during performing data transactions of workflow healthcare applications. Therefore, the proposed deep learning based blockchain technology is more in terms of above issues. Figure 4 (b,c,d) shows the data validation performance of the blockchain technologies, and their obtained objective function and results for workflow healthcare applications. However, still, blockchain technologies exploited different algorithms such as proof of work, proof of stake, and proof of credibility  that are consumed much more resources and incurred higher delays in their data validation and consensus models. The failure and independence among nodes during validation and failure have long delays for different transactions. Therefore, Fig. 4 (a,b,c,d) shows that the deep blockchain scheme optimizes all aspects during processing transactions among nodes compared to existing studies. The study implemented four baseline approaches that scheduled the workflow tasks on different computing nodes based on their pre-set requirements. Figure 5 shows that makespan performance workflow tasks (e.g., 1000) by exploiting different parallel and distributed scheduling schemes (e.g., baseline 1,4) and DRLBTS. The evaluation shows that DRLBTS outperformed all existing schemes, as shown in Fig. 5. Figure 5 and Fig. 6 show that the workflows of 1000 and 2000 tasks are scheduled with the different computing nodes and have different objective function (e.g., makespans) by using both initial and dynamic adaptation scheduling strategies during runtime. Figure 5 and Fig. 6 show that DRLBTS minimized the makespan of all workflows as compared to existing strategies. Figure 7 and Fig. 8 show that the workflows of 1000 and 2000 tasks are scheduled with the different makespans using both initial and deep learning strategies during runtime in the network. Figure 7 and Fig. 8 show that DRLBTS minimized the makespan of all workflows as compared to existing strategies. Figure 5, 6, 7, 8 shows the performances of baseline approaches as compared to the proposed scheme DRLBTS. There is the evaluation of different performances of healthcare workflow applications, such as makespan, failure rate, and deadline. Figure 5 shows the performances of different workflow strategies in terms of makespan (e.g., RPD%).  Model-free optimal policy-enabled scheduling proposed by the study reduces the mean-time delay of workflow applications compared to static and dynamic scheduling. The main reason is that the static and dynamic schedulers cannot handle uncertainty regarding resource capacity and leakage in the heterogeneous mobile fog cloud nodes. Figure 4 shows the effectiveness of deep learning-enabled blockchain for workflow healthcare applications is the more optimal in terms of resource leakage, data validation, failure, and inter-dependency, as shown in Fig. 4 (a), (b), (c) and (d) with the different proposed schemes. The existing ethereum static and dynamic Corda blockchain did not focus on resource leakage, failure, and workflow dependency in their model. Because these blockchain frameworks only considered the coarse-grained and fine-grained workloads in their models. Existing Ethereum static and dynamic Corda blockchain only validated single node type data with homogeneous nodes, and data offloads to servers only have standardized nodes.  Hence, all the simulation results show that, blockchain-based data validation and transaction consume much more resources and have higher delays in different computing nodes. It is practically impossible to obtain from all computing nodes to have the same resource capability and expect the same performance while implementing blockchain-based data validation schemes for the workflow applications. Therefore, the partitioning of workflow applications on the nodes and adaptive scheduling-based blockchain scheme that is proposed in this work gained more optimal results and minimized the overall makespan of applications, and handled all failure, resource leakage, and deadline of tasks and nodes as compared to existing frameworks and schemes.

## Finding and limitations

The study designed the DRLBTS algorithm scheme that divided the workflows into different tasks (mobile tasks, fog tasks and cloud tasks) and executed them on the different nodes. The divided tasks, such as mobile, fog, and cloud, are executed based on requirements resources, blockchain validation, and time for each application. However, there are many limitations in the proposed DRLBTS. The proposed DRLBTS consumes much energy while executing the workflows on different nodes. Therefore, in our future work, we will consider the different constraints such as energy consumption, electricity cost, and mobility-enabled power efficiency scheme for applications.

## Conclusion

With the cooperative multi-agents mechanism used in the study obtained the optimal results as shown in simulation part.  The study proposed DRLBTS algorithm framework that obtained the optimal results in terms of objective functions of the healthcare workflows as compared to existing methods. The current version of the study focuses on multi-agents in distributed mobile fog and cloud networks. The study showed that, DRLBTS executed the workflows on the different computing nodes, where the proposed deep blockchain scheme achieved the optimal delay transactions as compared to existing blockchain technologies. DRLBTS handles all situations, such as resource failure, task failure, resource leakage, and deadline.  However, this framework will not support real-time healthcare applications and will incur overhead at different nodes. Therefore, in future work, we will implement real-time profiling, which analyzes the application requirements at the runtime of applications. In future work, we will divide the application into parts based on the resources of each node and schedule them based on the quality of service they need. In future work, DRLBTS will optimize the energy consumption of the nodes and minimize the electricity cost during the execution of healthcare workflows in different time zones in distributed mobile fog cloud networks.

## Data Availability

The datasets generated and analyzed during the current study are not publicly available. The main reason is that we have designed self-workflows on the local machines and experimented with them on the local machines. Some of the datasets were gathered from local clinics. Therefore, they allowed only experiments that were not publicly available for analysis. The study designed the DRLBTS system based on Java programming for healthcare workflow applications. As the link below explains, the algorithm is based on deep reinforcement learning and blockchain technology. https://github.com/ABDULLAH-RAZA/Assignment-/tree/master.
